# Assessing Uncontrolled Confounding in Associations of Being Overweight With All-Cause Mortality

**DOI:** 10.1001/jamanetworkopen.2022.2614

**Published:** 2022-03-01

**Authors:** Maya B. Mathur, Tyler J. VanderWeele

**Affiliations:** Quantitative Sciences Unit, Stanford University, Palo Alto, California; Department of Pediatrics, Stanford University, Palo Alto, California; Department of Epidemiology, Harvard University, Boston, Massachusetts

## Introduction

Is being overweight associated with all-cause mortality, and if so, could the association simply be an artifact of uncontrolled confounding? This question has been controversial, with 2 prominent meta-analyses of nonrandomized studies reporting opposing conclusions. Flegal et al^1^ reported a protective association of being overweight (but not having obesity) vs having normal weight (hazard ratio [HR], 0.94 [95% CI, 0.91-0.96]), whereas the Global Body Mass Index (BMI) Mortality Collaboration^2^ (GBMC) reported a detrimental association (HR, 1.11 [95% CI, 1.10-1.11]). Just as individual nonrandomized studies can be biased because of uncontrolled confounding,^3^ so can meta-analyses.^4^ In both meta-analyses, many of the included studies did not control for probable confounders (eMethods in the Supplement). We investigated the extent to which potential uncontrolled confounding may have biased these meta-analyses' observed associations.

## Methods

To consider how strong uncontrolled confounders in each meta-analyzed study would have to be to negate the observed results, this reanalysis of meta-analyses applied sensitivity analyses for meta-analyses^4,5^ that are analogous to the E-value^3^ (analyses conducted on January 7, 2022). First, we calculated an E-value representing the minimum strengths of associations on the risk ratio (RR) scale that uncontrolled confounders would need to jointly have with being overweight and with mortality across all studies in each meta-analysis to shift the meta-analytic estimate or its 95% CI to the null (eMethods in the Supplement).

Second, we estimated the percentage of studies with meaningfully strong HRs (here defined as HR > 1.1 for associations in the detrimental direction and HR < 0.90 for associations in the protective direction), initially without considering potential uncontrolled confounding.^5,6^ Random-effects meta-analyses accommodate the possibility that studies’ underlying associations differ by estimating the distribution of these study-specific associations; this distribution can then be used to estimate the percentage of studies with meaningfully strong HRs (eMethods in the Supplement).^5,6^ If this percentage is large (eg, 80%), this may suggest that most studies have meaningfully strong HRs, albeit prior to considering uncontrolled confounding.^4,5^ We then assessed how strong uncontrolled confounding would have to be to reduce the percentage of meaningfully strong HRs to 15%.^5^ For code and data for reproducibility, see eMethods in the Supplement.

## Results

Before considering uncontrolled confounding, our reanalysis obtained pooled HRs of 0.93 (95% CI, 0.91-0.96; *P* < .001; heterogeneity $\hat{\tau }=0.13$) for Flegal et al^1^ and 1.10 (95% CI, 1.07-1.12; *P* < .001; $\hat{\tau }=0.08$) for GBMC.^2^ These analyses comprised, respectively, 140 and 186 estimates from prospective nonrandomized cohorts.

For Flegal et al,^1^ the E-value suggested that uncontrolled confounders that were associated with being overweight and with lower mortality by an RR of 1.36 each could suffice to shift the point estimate to the null. For GBMC,^2^ uncontrolled confounders that were associated with being overweight and with mortality by an RR of 1.43 each could suffice to shift the point estimate to the null. To shift each CI so that it included the null, the analogous confounding associations in Flegal et al^1^ and GBMC^2^ would be RRs of 1.25 and 1.36, respectively.

Before considering uncontrolled confounding, we estimated the percentage of studies having meaningfully strong protective HRs (ie, <0.9) as 40% (95% CI, 28%-51%) in Flegal et al^1^ but 0% in GBMC.^2^ Conversely, we estimated that 50% (95% CI, 34%-63%) of studies in GBMC^2^ but 9% (95% CI, 4%-15%) of studies in in Flegal et al^1^ would have meaningfully strong detrimental HRs (ie, >1.1). Again, these percentages refer to the heterogeneous distribution of associations that underlie each meta-analysis. However, small uncontrolled confounding associations (ie, confounding RRs of approximately 1.43 for Flegal et al^1^ and 1.37 for GBMC^2^) would suffice for both meta-analyses to bring the percentages of causal effects with HRs below 0.9 (for Flegal et al^1^) or above 1.1 (for GBMC^2^) to the same level (15%) in the 2 meta-analyses (Figure).

## Discussion

This reanalysis of meta-analyses found that for 2 meta-analyses, uncontrolled confounding associations in each study with RRs of 1.25 to 1.43 could potentially shift the point estimate or CIs to the null or substantially decrease the percentage of meaningfully strong effect sizes. These small degrees of uncontrolled confounding seem plausible in the context of studies’ limited control of confounding by physical, social, behavioral, and psychological factors. These sensitivity analyses suggest that neither meta-analysis provided robust evidence for protective or detrimental potential effects of being overweight on mortality.

Our analysis has several limitations. First, we considered bias of constant severity across studies. Second, thresholds defining associations with meaningfully large HRs (eg, HR > 1.1) are somewhat arbitrary and should be informed by scientific context.^5,6^ We found that uncontrolled confounding may temper interpretation of meta-analyses. The differing results of Flegal et al^1^ and GBMC^2^ may reflect heterogeneous effects and differing inclusion criteria, along with differing uncontrolled confounding. Establishing potentially modest effects of being overweight on mortality would require improved study designs for primary studies and meta-analyses alike.^4^

## Supplementary Material

1

## Figures and Tables

**Figure.**
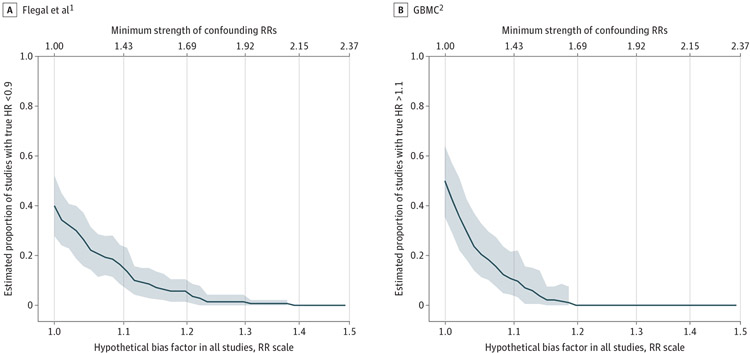
Estimated Proportions of Meaningfully Strong Effect Sizes The estimated proportions of meaningfully strong effect sizes in each meta-analysis as a function of hypothetical confounding bias in each meta-analyzed study are presented. The x-axis is presented on the log scale, with tick marks on the risk ratio (RR) scale. GBMC indicates Global Body Mass Index (BMI) Mortality Collaboration; HR, hazard ratio; shaded areas, 95% pointwise CIs, which are omitted when they were not statistically estimable (ie, for percentages close to 0).
