# Supplementary material for: Assessing Uncontrolled Confounding in Associations of Being Overweight With All-Cause Mortality
Source: JAMA Netw Open. 2022 Mar 28;5(3):e222614. doi: 10.1001/jamanetworkopen.2022.2614 (PMC8961316; doi:10.1001/jamanetworkopen.2022.2614)
Supplement: Supplementary file 1 [file jamanetwopen-e222614-s001.pdf]

Supplemental Online Content

Mathur MB, VanderWeele TJ. Assessing uncontrolled confounding in associations of being overweight with all-cause mortality. *JAMA Netw Open*. 2022;5(3):e222614. doi:10.1001/jamanetworkopen.2022.2614

eMethods.
eReferences.

This supplemental material has been provided by the authors to give readers additional information about their work.

© 2022 Mathur MB et al. *JAMA Network Open.*

eMethods.

1.1. The E-value

The E-value for a single study represents the minimum strength of association, on the risk ratio (*RR*) scale, that uncontrolled confounder(s) would need to have with the exposure and/or the outcome, conditional on any measured and controlled confounders, to "explain away" (or negate) an observed association or effect.[1,2] It is equivalent to interpret the E-value as the minimum strengths of association that uncontrolled confounder(s) would need to have with *both* the exposure and the outcome, if these 2 strengths of association are taken to be of equal magnitude. We adopt the latter interpretation in the main text, describing "joint" associations as a shorthand. E-values for confidence intervals similarly represent the minimum strength of association, on the risk ratio (*RR*) scale, that uncontrolled confounder(s) would need to have with the exposure and/or the outcome, conditional on any measured and controlled confounders, to shift the effect size such that the confidence interval includes the null.[1,2]

Details and reporting guidelines for the standard E-value are discussed elsewhere.[1-4] The E-value has limitations, which have been discussed and debated elsewhere.[5-8] The same considerations and limitations regarding interpretation apply to the meta-analysis analogs wediscuss here.

© 2022 Mathur MB et al. *JAMA Network Open.*

1.2. The percentage of meaningfully strong effect sizes

Random-effects meta-analyses have reported the percentage of studies with meaningfully strong effect sizes to characterize evidence strength across numerous studies with effect sizes or associations that may differ.[9-11] That is, by conducting a random-effects meta-analysis, the meta-analyst acknowledges the possibility that studies' effects differ (e.g., due to differences in their populations) by assuming that these effect sizes come from a distribution that might be highly concentrated around the meta-analysis mean (i.e., low heterogeneity) or alternatively could be more spread out (i.e., high heterogeneity).The meta-analytic pooled estimate represents the mean of this distribution. As a supplement to the meta-analytic estimate, the percentage of meaningfully strong effect sizes helps assess, in the potentially heterogeneous distribution of effect sizes, how often those effect sizes are meaningfully strong. If this percentage is large (e.g., 80%), this would suggest meaningfully strong associations in most studies, albeit prior to considering potential bias due to uncontrolled confounding.[12,13] Then, as a sensitivity analysis to consider confounding, one can ask: "How strong would the potential influence of uncontrolled confounder(s) have to be to reduce this percentage of meaningfully strong effect sizes to below a certainthreshold?"

1.3. Confounding control in meta-analyzed studies

In Flegal et al.'s[15] meta-analysis, approximately half of the studies adjusted for age, sex, and smoking; Flegal et al.[15] reported similar results when analyzing only studies that did control for these variables. GBMC[14] restricted their analysis to individual participants who were never-smokers without specific chronic diseases, controlled within each study for age and sex, and omitted the first 5 years of follow-up (when these data were available). Omitting the first 5 years of follow-upcould, in principle, reduce confounding by underlying health conditions, but this method does havesubstantial limitations, essentially because early mortality may be a weak surrogate for the presenceof underlying health conditions.[16,17] In both meta-analyses, most (or all) studies did not adjust for probable confounders such as socioeconomic status, physical activity, dietary quality, and baselinebody mass index (BMI).

1.4. Methods for re-analysis and primary sensitivity analyses

We conducted all data analyses using R statistical software version 4.0.2 (R Project for Statistical Computing). We conducted analyses from December 2021 to January 2022 using data sets provided by the meta-analysts at our request. All *P* values are 2-tailed. To conduct the sensitivity analyses, we first obtained point estimates and confidence intervals by fitting a standard random-effects meta-analysis by restricted maximum likelihood and with standard errors estimated with the Knapp-Hartung adjustment.[18] Throughout, we treated hazard ratios as approximately equal to risk ratios because the outcome was rare.

In both meta-analyses, some papers, consortia, or cohorts contributed multiple point estimates.

© 2022 Mathur MB et al. *JAMA Network Open.*

In other cases, multiple cohorts were pre-aggregated into a single estimate (see Supplements of references[14-15]). Throughout the main text and this Supplement, we use "studies" to refer to the meta-analyzed point estimates. This terminology differs from that used in the meta-analyses themselves, such that we report different numbers of "studies" (140 for Flegal et al.[15] and 186 for GBMC[14]) than were reported in the original meta-analyses (97 and 189, respectively). Because the original analyses did not seem to account for clustering of estimates within papers, consortia, or cohorts, we similarly analyzed both datasets using a simple random-effects model that assumed independent estimates. (GBMC's[14] analysis accounted for cohorts' contributing multiple outcomes that represented different BMI contrasts, but this is distinct from clustering of estimates for a single BMI contrast within, for example, a consortium.) However, note that a best-practice meta-analysis would account for the clustering via, for example, robust estimation[19] or multilevel modeling, or a combination.[20]

GBMC[14] conducted several analyses, for example by defining BMI categories at different levelsof granularity. For comparability to Flegal et al.'s [15] meta-analysis using standard BMI categories, our re-analysis of GBMC's data used the standard BMI range for being overweight (i.e., $25 \leq \text{BMI} < 30$). Additionally, GBMC's[14] analyses considered dose-response across BMI categories, so used multivariate meta-analysis and floating variance estimates to account for the multiple BMI categories contributed by each cohort. Again for comparability to Flegal et al.'s[15] meta-analysis and because we focused on only one contrast in BMI categories (i.e., being overweight vs. being normal weight), we used standard univariate meta-analysis methods and inference rather than multivariatemeta-analysis. Because of these methodological differences, our point estimate and confidence interval for GBMC[14] differed negligibly from their reported $HR = 1.11$ (95% CI: [1.10, 1.11]) for their analysis that used standard BMI categories.

We calculated E-values for the point estimate and confidence interval using methods and software that have been described elsewhere.[21.22] We estimated the percentage of meaningfully strong effect sizes (as defined in the main text) using nonparametric methods when considering bias of homogeneousstrength across studies.[13] As described in the main text, we considered effect sizes to be meaningfully strong when they were greater than $HR = 1.1$ (for estimates in the apparently detrimental direction) or when they were less than $HR = 0.9$ (for estimates in the apparently protective direction), such that being overweight confers at least a 10% increased or decreased hazard of mortality. These choices are of course somewhat arbitrary, but analyses with other thresholds yielded similar conclusions about sensitivity to uncontrolled confounding. Suggestions for how to choose such thresholds have been discussed elsewhere.[21,23] Our sensitivity analyses considered the strength of uncontrolled confounding associations that would be required to reduce the percentage of meaningfully strong effect sizes to less than 15%, a criterion we chose based on previous recommendations.[21]

1.5. Reproducibility

All R code required to reproduce these results is publicly available (https://osf.io/b3ux8/). Data from the two meta-analyses cannot be made public at the authors' request, but they are available upon request to individuals who have secured permission from the original authors.

© 2022 Mathur MB et al. *JAMA Network Open.*

eReferences.

[1] Tyler VanderWeele and Peng Ding. Sensitivity analysis in observational research: introducing the E-value. *Annals of Internal Medicine*, 167(4), 268-274.

[2] Peng Ding and Tyler J VanderWeele. Sensitivity analysis without assumptions. *Epidemiology*, 27(3):368, 2016.

[3] Tyler J VanderWeele, Peng Ding, and Maya Mathur. Technical considerations in the use of the E-value. *Journal of Causal Inference*, 7(2), 2019.

[4] Tyler J VanderWeele and Maya B Mathur. Commentary: developing best-practice guidelines for the reporting of E-values. *International Journal of Epidemiology*, 49(5):1495–1497, 2020.

[5] Charles Poole. Commentary: Continuing the E-value's post-publication peer review. *International Journal of Epidemiology*, 49(5):1497–1500, 2020.

[6] Sander Greenland. Commentary: An argument against E-values for assessing the plausibility that an association could be explained away by residual confounding. *International Journal of Epidemiology*, 49(5):1501–1503, 2020.

[7] John PA Ioannidis, Yuan Jin Tan, and Manuel R Blum. Limitations and misinterpretations of E-values for sensitivity analyses of observational studies. *Annals of Internal Medicine*, 170(2):108–111, 2019.

[8] Tyler J VanderWeele. Are Greenland, Ioannidis and Poole opposed to the Cornfield conditions? A defence of the E-value. *International Journal of Epidemiology*, 2021, in press.

[9] Sebastian E Baumeister, Michael F Leitzmann, Jakob Linseisen, and Sabrina Schlesinger. Physical activity and the risk of liver cancer: a systematic review and meta-analysis of prospective studies and a bias analysis. *JNCI: Journal of the National Cancer Institute,* 111(11):1142–1151, 2019.

[10] Joseph GL Lee, Amanda Y Kong, Kerry B Sewell, Shelley D Golden, Todd B Combs, Kurt M Ribisl, and Lisa Henriksen. Associations of tobacco retailer density and proximity with adult tobacco use behaviours and health outcomes: A meta-analysis. *Tobacco Control*, 2021.

[11] Guo-Qiang Zhang, Jin-Liang Chen, Ying Luo, Maya B Mathur, Panagiotis Anagnostis, Ulugbek Nurmatov, Madar Talibov, Jing Zhang, Catherine M Hawrylowicz, Mary Ann Lumsden, et al. Menopausal hormone therapy and women's health: An umbrella review. *PLoS Medicine*, 18(8):e1003731, 2021.

[12] Maya B Mathur and Tyler J VanderWeele. Methods to address confounding and other biases in meta-analyses: Review and recommendations. *Annual Review of Public Health*, 2022, in press. Preprint retrieved from https://osf.io/v7dtq/.

[13] Maya B Mathur and Tyler J VanderWeele. Robust metrics and sensitivity analyses for meta-analyses of heterogeneous effects. *Epidemiology*, 31(3):356–358, 2020.

© 2022 Mathur MB et al. *JAMA Network Open.*

[14] Global BMI Mortality Collaboration. Body-mass index and all-cause mortality: individual participant-data meta-analysis of 239 prospective studies in four continents. *The Lancet*, 388(10046):776–786, 2016.

[15] Katherine M Flegal, Brian K Kit, Heather Orpana, and Barry I Graubard. Association of all-cause mortality with overweight and obesity using standard body mass index categories: a systematic review and meta-analysis. *Journal of the American Medical Association*, 309(1):71–82, 2013.

[16] David B Allison, Moonseong Heo, Dana W Flanders, Myles S Faith, and David F Williamson. Examination of "early mortality exclusion" as an approach to control for confounding by occult disease in epidemiologic studies of mortality risk factors. *American Journal of Epidemiology*, 146(8):672–680, 1997.

[17] David B Allison, Moonseong Heo, Dana W Flanders, Myles S Faith, Kenneth M Carpenter, and David F Williamson. Simulation study of the effects of excluding early deaths on risk factor-mortality analyses in the presence of confounding due to occult disease: the example of body mass index. *Annals of Epidemiology*, 9(2):132–142, 1999.

[18] Guido Knapp and Joachim Hartung. Improved tests for a random effects meta-regression with a single covariate. *Statistics in Medicine*, 22(17):2693–2710, 2003.

[19] Larry V Hedges, Elizabeth Tipton, and Matthew C Johnson. Robust variance estimation in meta-regression with dependent effect size estimates. *Research Synthesis Methods*, 1(1):39–65, 2010.

[20] James E Pustejovsky and Elizabeth Tipton. Meta-analysis with robust variance estimation: Expanding the range of working models. *Prevention Science*, 2021, in press.

[21] Maya B Mathur and Tyler J VanderWeele. Sensitivity analysis for unmeasured confounding in meta-analyses. *Journal of the American Statistical Association*, 115(529):163–172, 2020.

[22] Maya B Mathur, Peng Ding, Corinne A Riddell, and Tyler J VanderWeele. Website and R package for computing E-values. *Epidemiology*, 29(5):e45, 2018.

[23] Maya B Mathur and Tyler J VanderWeele. New metrics for meta-analyses of heterogeneous effects. *Statistics in Medicine*, 38(8):1336–1342, 2019.

© 2022 Mathur MB et al. *JAMA Network Open.*
